# Voluntary Hospital Finance. The Position and Outlook Better

**Published:** 1919-11-08

**Authors:** 


### The Hospital

The Workers' Newspaper of Administrative Medicine and Institutional Life, Administration, National Insurance and Health.

No. 1744, Vol. LXVII. SATURDAY, NOVEMBER 8, 1919. PRICE TWOPENCE

### VOLUNTARY HOSPITAL FINANCE.
### THE POSITION AND OUTLOOK BETTER.

Since we published the article "A Treasured Remembrance and Great Opportunity" in our last issue, circumstances have arisen which have confirmed the soundness of the policy then expounded. Facts have been brought to our notice which show clearly that the life-blood of a number of the hospitals is at the mercy of individuals who apparently lack the courage and the outlook to understand the present pressing war-caused emergencies, or the strength and will to attempt to overcome them. The circumstances are that a large portion of the Press, in season and out of season, continuously pressed for and insisted upon the preparation and issue of a second Budget before Christmas, with further taxation to raise at once a great sum towards the liquidation of the war debt which burdens the nation. These demands were accompanied by insistence that if this was not done bankruptcy for the nation was ahead. Every effort was made to move Parliament to defeat the Government and cast everything into the melting-pot. This violent crusade was carried on against the Government and the classes which, despite their continuous and zealous efforts and work, have barely been able to maintain their families and households in comfort and retain the small margin of reserve which was left them after meeting existing heavy taxation. The head of the section of the Press which conducted this crusade had, it was realised, by some previous agitations, in various directions, produced revolutionary results, and might end in reducing to a state verging on bankruptcy many thousands of middle-class workers and others, who collectively, from the State's point of view, represented the most thrifty, philanthropic and reliable classes of bread-earners and workers. Some of the most able, hard-working and considerable bread-earners tried by shortening their hours of sleep, and undertaking special and additional work, thus to add to their income. They found that when the expenses of necessary assistance and other charges were paid, and the income-tax and rates, etc., were met, no appreciable portion remained for the man who had done the work and been paid for it. We know cases indeed where hundreds of pounds' worth of work, and say two hours of lessened sleep several days a week, resulted to this type of worker in no appreciable addition to his available resources to enable him to pay his way and support his family. This experience was not confined, we found, to any special class of worker but, in the later war conditions, has suspended the earning power and income of successful business men of keen intelligence and business gifts.

Such was the outlook before the commencement of the Finance Debate in the House of Commons on Wednesday, October 29. The debate and the division which followed, whereby the Government was left with a majority of 355 votes—by which number they defeated an amendment, for which 50 members voted—swept away and reduced to Parliamentary dust and ashes the Press work of destruction so strenuously pursued by the opponents of the Prime Minister. The Government motion was then agreed to *nem. con.*, and members of the House of Commons thereby promised its "hearty support to the Government in all reasonable proposals, however drastic, for the reduction of expenditure and the diminution of debt." Thus the atmosphere was cleared of many fears which had brought sleepless nights and endless worries to a large section of real workers within the nation, for they were thus relieved from the threat of more and heavy taxation before the year was out, and could turn to their own affairs and budgets with a quietness and hope which they had previously lacked under the circumstances mentioned above. This large section of workers included some of the most generous and continuous givers to our philanthropic institution which the country contains. There can be no doubt that very many of them, when they make up their accounts for the year, will have in their mind the greater need and enforced increased expenditure imposed on our greater hospitals. As they breathe again with hope in the future they will cherish the wish, that the burden of taxation which they have individually borne in silence, almost to the breaking-point. may be stayed by the institution of a wiser policy of finance, and a reform and remodelling of the archaic accountancy and old-fashioned system under which the Treasury's book-keeping in existing conditions has proved its inadequacy, and revealed the mass of misconceptions and faulty estimates which have disturbed His Majesty's lieges in the immediate past.

The action of the House of Commons then, there is solid ground to hope, may not only have relieved the political crisis, but ensured that those hospitals in our midst, which are managed by people with wise heads, untiring industry, and a just belief in the hold the voluntary system in this country deservedly has on the goodwill and confidence of all classes, may find their budgets, if wisely framed on right lines, will be lightened, and the cry of the pessimists and unbelievers drowned in the welcome fact, that the income of the current year, where adequate brains, energy, and devotion have been at the helm, will prove adequate to meet the current year's expenditure, and may improve the hospital revenues permanently too. So far well. How many hospitals will be wise enough to make the supreme effort and follow the lines expounded in our last week's issue?

We must defer until next week the explanation why the life-blood of a number of hospitals is largely dependent on individuals who must strengthen their faith, and devote themselves entirely to business; or, failing this, retire from hospital work and seek new fields for their attenuated energies. We have given above a curious illustration of what we consider blind legislation, enforced by statute, without adequate and precise knowledge of its probable effects, before destructive imposts find a place on the statute book, has already resulted in this country. We have been approached by editors of important lay papers, who are a little confused by the unworthy and ill-founded assertion of certain hospital workers, insufficiently informed in the actual position and outlook for voluntary hospital finance in this country to-day. We hope, however, that such utterances will not be taken too seriously, and that they will certainly not prevent our lay colleagues in charge of big newspapers from lending a hand to arouse the nation's and hospital authorities, whom it will immediately affect, to the necessity for waking up and setting to work in the financial sense with a knowledge and a will adequate to the heavier tasks which the voluntary hospitals have to face at the moment with British pluck and resolution. That the British public will respond to those who adopt this course is demonstrated by past experience, and all wisely administered institutions throughout the country should soon find themselves in possession of greater influence and lessened anxiety for the future.

